# Soluble Platelet Release Factors as Biomarkers for Cardiovascular Disease

**DOI:** 10.3389/fcvm.2021.684920

**Published:** 2021-06-21

**Authors:** Gaukhar Baidildinova, Magdolna Nagy, Kerstin Jurk, Philipp S. Wild, Hugo ten Cate, Paola E. J. van der Meijden

**Affiliations:** ^1^Departments of Biochemistry and Internal Medicine, Cardiovascular Research Institute Maastricht (CARIM), Maastricht University, Maastricht, Netherlands; ^2^Center for Thrombosis and Hemostasis, University Medical Center of the Johannes Gutenberg-University Mainz, Mainz, Germany; ^3^DZHK (German Center for Cardiovascular Research), Partner Site RhineMain, Mainz, Germany; ^4^Preventive Cardiology and Preventive Medicine, Center for Cardiology, University Medical Center of the Johannes Gutenberg-University Mainz, Mainz, Germany; ^5^Thrombosis Expertise Center, Heart and Vascular Center, Maastricht University Medical Center, Maastricht, Netherlands

**Keywords:** platelets, biomarkers, thrombosis, venous thromboembolism, atrial fibrillation, arterial thrombosis

## Abstract

Platelets are the main players in thrombotic diseases, where activated platelets not only mediate thrombus formation but also are involved in multiple interactions with vascular cells, inflammatory components, and the coagulation system. Although *in vitro* reactivity of platelets provides information on the function of circulating platelets, it is not a full reflection of the *in vivo* activation state, which may be relevant for thrombotic risk assessment in various disease conditions. Therefore, studying release markers of activated platelets in plasma is of interest. While this type of study has been done for decades, there are several new discoveries that highlight the need for a critical assessment of the available tests and indications for platelet release products. First, new insights have shown that platelets are not only prominent players in arterial vascular disease, but also in venous thromboembolism and atrial fibrillation. Second, knowledge of the platelet proteome has dramatically expanded over the past years, which contributed to an increasing array of tests for proteins released and shed from platelets upon activation. Identification of changes in the level of plasma biomarkers associated with upcoming thromboembolic events allows timely and individualized adjustment of the treatment strategy to prevent disease aggravation. Therefore, biomarkers of platelet activation may become a valuable instrument for acute event prognosis. In this narrative review based on a systematic search of the literature, we summarize the process of platelet activation and release products, discuss the clinical context in which platelet release products have been measured as well as the potential clinical relevance.

## Introduction

Platelet thrombus formation is a process of crucial importance in hemostasis and thrombosis, starting with platelet activation, adhesion, and aggregation at the vessel wall surface that is damaged by trauma, inflammation, or, in case of atherosclerosis, altered by an atherosclerotic plaque (1, 2). In general, upon vascular damage the platelet membrane glycoprotein (GP) Ib/V/IX complex interacts with von Willebrand factor (vWF) from the damaged endothelium leading to the adhesion of platelets (2, 3). Tethered platelets bind to collagen through their GPVI and integrin α_2_β_1_ receptors, which potently trigger platelet activation. The activation process continues toward the release of soluble mediators from activated platelets, an increase of cytosolic Ca^2+^, and the formation of a platelet thrombus. In parallel, fibrin formation is triggered by the tissue factor-driven-coagulation cascade and amplified by thrombin-mediated feedback reactions as well as the contact activation pathway. Platelet and coagulation activation are highly intertwined with multiple interactions between these two processes. Not only is thrombin a key mediator of platelet activation, platelets also promote coagulation *via* phosphatidylserine exposure and receptor-mediated binding of coagulation factors (4).

Upon activation, platelets release more than 300 proteins, including P-selectin (CD62P), CD40 ligand (CD40L), platelet factor 4 (PF4), and many others (5). Some of these platelet release markers can reflect the *in vivo* platelet activation status and hence have already been investigated in clinical studies addressing the involvement of platelet activation in patients with different thrombotic diseases. The soluble platelet biomarkers may provide a better way of assessing the thrombotic risk than the conventional platelet function tests. Impaired *in vitro* platelet activation based on platelet function tests, may on the one hand point to dysfunctional platelets, but on the other hand to prior activation in the circulation potentially resulting in an exhausted platelet phenotype. Therefore, soluble platelet activation markers reveal the *in vivo* platelet activation status and provide information on the underlying pathophysiological mechanisms in thrombosis-related disease (6).

The role of platelets in atherothrombotic disease, characterized by arterial thrombus formation as a consequence of atherosclerotic lesion disruption, is well-established. Vascular occlusion underlies the occurrence of ischemia in specific vascular beds, resulting in coronary artery disease (CAD), myocardial infarction (MI), peripheral artery disease (PAD), and ischemic stroke (IS) (7). Although in arterial thromboembolism—as a consequence of atrial fibrillation—and venous thrombosis, coagulation activation is the predominant process, accumulating evidence demonstrates pathogenic roles of platelets herein (8). The conventional treatment strategy for atherothrombotic disease and arterial/venous thromboembolism is based on antiplatelet and anticoagulant drugs, respectively. Especially for patients with atherothrombotic events, the combined antiplatelet and anticoagulant treatment appears beneficial and has recently gained more attention (9).

The active participation of platelets in cardiovascular diseases and the established fact that antiplatelet therapy decreases the risk of (recurrent) thrombotic events underlines the importance of research in platelet pathophysiology (10). In this narrative review based on a systematic search of the literature, we summarized the process of platelet activation and release products, discuss the clinical context in which platelet release products have been measured as well as the potential clinical relevance. Here we focus on soluble platelet biomarkers in patients with arterial thrombosis, venous thrombosis, and atrial fibrillation.

## Platelet Release Factors

Activated platelets release small biomolecules and more than 300 proteins, which regulate hemostatic, inflammatory, and angiogenic responses of platelets, leukocytes, and vascular cells. Major sources of the platelet protein releasate are granule cargos and proteolytically cleaved/shed membrane-bound proteins such as receptors and platelet-derived extracellular vesicles. Advanced enzyme-linked immunosorbent assay (ELISA)-based assays and mass spectrometry approaches enable the qualitative and quantitative assessment of platelet-released proteins in plasma and of isolated platelets, respectively (11). Platelets contain three major types of granules: α-granules, dense or δ-granules, and lysosomes (12, 13). Rapid granule release can be induced by diverse agonists like thrombin, collagen, and their related peptides (11). The platelet α-granule secretome covers the majority of released platelet proteins, which are synthesized in megakaryocytes or endocytosed from plasma. The α-granules contain large adhesive proteins [vWF, thrombospondin-1 (TSP-1), vitronectin, fibronectin], coagulation factors (factor V, VII, XI, XIII), mitogenic factors [platelet-derived growth factor (PDGF), vascular endothelial growth factor (VEGF), transforming growth factor β (TGF-β)], protease inhibitors [protein C, plasminogen activator inhibitor 1 (PAI-1), tissue factor pathway inhibitor (TFPI)], membrane proteins [P-selectin (CD62P), CD40L], chemokines [β-thromboglobulin (beta-tg), PF4, Regulated upon Activation, Normal T-Cell Expressed and Presumably Secreted (RANTES), stromal cell-derived factor-1α (SDF-1α)], and several other molecules, which are released immediately upon platelet activation (14). Activated platelets also release thromboxane A2 (TxA2), a product of arachidonic acid metabolism (15), and several other eicosanoids (16).

In contrast, dense granules secrete small soluble molecules, such as serotonin, glutamate, adenosine diphosphate (ADP), adenosine triphosphate (ATP), histamine, polyphosphate, Ca^2+^, and Mg^2+^ (17, 18). Together with TxA2, they function as positive feedback mediators of platelets to promote platelet aggregation and platelet-based coagulation. Platelet-derived serotonin promotes thrombosis development by inducing vasoconstriction and enhancing platelet activation and thrombus formation. The platelet lysosomes contain enzymes required for extracellular matrix degradation, cell migration, antimicrobial activity, and thrombus remodeling (19, 20). Among these enzymes are cathepsin D and E, β-hexosaminidase, elastase, and heparanase (11). The classical flow cytometry protein for dense granule and lysosomal membrane detection is CD63.

In addition to the release of soluble proteins from granules, proteolytic cleavage of platelet membrane proteins occurs mainly by metalloproteinases (MMP) and the shed fragments can modulate cellular responses. The platelet sheddome, excluding plasma proteins and platelet-derived extracellular vesicles, contains at least 69 membrane proteins (21, 22). Only a fraction of all membrane proteins is cleaved, among these are the externalized surface proteins P-selectin and CD40L, the receptor GPIbα, GPV subunits of the GPIb-IX-V complex, and GPVI (21, 23–25). The ectodomains of the receptors are shed in response to ligand engagement, elevated shear, coagulation, or apoptosis.

### Platelet Secretome

Upon activation, platelets secrete beta-tg from α-granules that are derived from the proteolytic cleavage of platelet basic proteins resulting in CTAPIII, CXCL7, and beta-tg. Beta-tg shares significant homology with PF4 (26). Both molecules belong to the chemokine CXC subfamily (27) and are expressed in monocytes, granulocytes, T-cells, and mast cells (7). Yet, platelets have been proposed as the primary and the most rapidly available source of the aforementioned chemokines (28). Beta-tg accounts for almost 10% of the α-granules content and is released into the blood with PF4 and other proteins upon platelet activation (27). The half-life of beta-tg in the blood is 100 min (29), depending on renal clearance (30, 31), while PF4 is rapidly cleared by binding to endothelial cells (32).

Another chemokine secreted by platelet α-granules is SDF-1α or C-X-C motif chemokine ligand 12 (CXCL12), which is involved in inflammatory pathways. SDF-1α is expressed by various cells throughout the body, including immune, stem, and endothelial cells (33), but platelets are thought to be the primary source. Following platelet activation, SDF-1α remains surface-bound and a strong stimulus is required to mediate release. ADP stimulation appears to be most potent in inducing SDF-1α release. Although SDF-1α in the circulation is susceptible to proteolytic degradation, it might be protected in the microenvironment of platelet thrombi (34). There is evidence that SDF-1α *via* its chemokine receptor CXCR4 induces TxA2 production and dense granule release, which altogether contributes to thrombus formation (35). In addition, ligation of SDF-1α to CXCR4 and CXCR7 regulates monocyte function and macrophage/foam cell differentiation, indicating an important role of SDF-1α in inflammation (36).

TSP-1 is a high-molecular multidomain glycoprotein expressed by various cell types including endothelial cells, monocytes, macrophages, fibroblasts, smooth muscle cells, dendritic cells, and B-cells (37). Similar to the previously mentioned proteins, the main source of TSP-1 is platelets, where it is one of the most abundant granule proteins, synthesized by megakaryocytes. After platelet activation, TSP-1 is released from the α-granules and found either bound to the platelet membrane or in its soluble form in plasma. TSP-1 has multiple functions in hemostasis, angiogenesis, proliferation, migration, endocytosis, immune reactions, and apoptosis. In addition to vWF, TSP-1 has been identified as a high shear substrate for human platelets (38). The TSP-1-CD36 interaction promotes thrombus formation and stabilization under high shear conditions (39). Platelet-originated TSP-1 suppresses the activity of several proteases, amongst others, MMP-2 and−9, plasmin, and cathepsin G. TSP-1-deficient mice models were characterized by improper thrombosis and extended bleeding time (40).

VWF is a multimeric glycoprotein present in platelet α-granules and in Weibel-Palade bodies of endothelial cells (41). Weibel-Palade bodies secrete vWF continuously, but the amount of released vWF can be greatly increased in response to inflammatory stimuli. Since vWF is mainly secreted by endothelial cells, it is a marker for endothelial cell activation rather than platelet activation. After secretion, vWF multimers are cleaved by a disintegrin and metalloproteinase with a thrombospondin type 1 motif, member 13 (ADAMTS13), which is essential for maintaining normal hemostasis. At sites of vascular damage, collagen-bound vWF binds to the platelet GPIbα-IX-V complex and mediates platelet adhesion, especially under high shear conditions occurring in the arterial system. Furthermore, vWF functions as a carrier protein for coagulation factor VIII in the circulation. There are several reviews discussing the role of vWF in platelet activation and inflammation bringing vWF fame of a risk factor for both arterial and venous thrombosis (42–44).

Activated platelets also produce several eicosanoids, including TxA2, prostaglandin (PG) D_2_, PGE_2_, 11-, 12-, and 15-hydroxyeicosatraenoic acid (HETE), through arachidonic acid metabolism by the cyclooxygenase and lipoxygenase pathways (16). TxA2 is synthesized by platelets as well as endothelial cells, macrophages, and neutrophils (45). *Via* both autocrine and paracrine mechanisms, TxA2 stimulates platelet activation and further aggregation (46, 47). The half-life of TxA2 is about 30 s, therefore it cannot be measured under physiological conditions (46). However, the stable TxA2 metabolite thromboxane B2 (TxB2) has a half-life of 5–7 min and can be assessed by mass spectroscopy, liquid chromatography, and ELISA. A more common approach is to determine the level of TxA2 by measuring the TxB2 urine metabolite 11-dehydrothromboxane B2 (11-DH-TxB2).

PGD_2_ is mostly released by macrophages, but also to some extent by platelets, and is assumed to be a platelet activation inhibitor. PGE_2_ is mainly synthesized by endothelial cells and to a lesser extent by platelets. The effect of PGE_2_ on platelets is concentration-dependent; at low concentrations, it enhances platelet aggregation, while it inhibits aggregation at higher concentrations. 12-HETE is mainly produced by platelets but its effect on platelet activity is not fully investigated (48).

### Platelet Sheddome

While the platelet secretion markers are rapidly released upon platelet activation, receptor shedding *in vitro* requires strong platelet stimulation for a prolonged time (6). Shedding results in a soluble shed fragment and a remnant platelet-bound fragment and hence in the loss of receptor-ligand binding function (49). Especially the proteolytic release of GPIbα, GPV, and GPVI has been thoroughly investigated in the last decades.

GPIbα and GPV are part of the GPIb-IX-V complex, expressed exclusively in platelets and megakaryocytes (50) and critical for vWF-dependent platelet adhesion (2). Upon platelet activation, GPIbα shedding is dependent on a disintegrin and metalloproteinase (ADAM)17 activity, whereas GPV can be cleaved by ADAM10/17 and thrombin (51). ADAM17 has a decisive role in GPIbα shedding, determining 90% of the glycocalicin plasma levels, whereas ADAM10 deficiency has no impact on GPIbα shedding. Shear, oxidative stress, serotonin, and GPVI agonists are prominent triggers for ADAM17-mediated GPIbα shedding, resulting in the soluble ectodomain glycocalicin (51, 52). It is hypothesized that glycocalicin is able to trigger hepatic thrombopoietin production *in vivo* (53), however, the exact role of glycocalicin remains an object of further explorations. In the case of GPV, the main regulator of the shedding process is thrombin and it results in complete elimination of GPV from the platelet surface.

GPVI is another platelet lineage-specific molecule and it functions as a receptor for collagen and fibrin among others (54). The ectodomain shedding is differently regulated by ADAM10 and 17, and the intact receptor is released as a soluble fragment (55). Physiological agonists leading to GPVI shedding are collagen, fibrin, shear stress, antiplatelet autoantibodies, and factor Xa (52). The time frame of GPVI release is dependent on the potency of agonists and, for example, convulxin results in experiments in faster shedding than collagen. The platelet specificity of these receptors makes GPIbα, GPV, and GPVI attractive candidates for identifying platelet activation *in vivo*.

Contrary to GPVI and the GPIb-IX-V complex, ADAM10/17 are not involved in the shedding of the C-type lectin-like receptor 2 (CLEC-2) upon platelet activation (56). CLEC-2 is abundantly expressed in platelets and megakaryocytes and not in other blood cells (57), albeit a small amount of CLEC-2 is present in liver Kupffer cells (58). Soluble CLEC-2 (sCLEC-2) is shed as a small fragment or could be released bound to other platelet microparticles (56, 59), whereas sGPVI is always shed as a separate fragment. To date, only podoplanin has been recognized as a physiological ligand for CLEC-2 (57). Mouse studies indicate that CLEC-2 has only a minor role as an adhesion receptor in hemostasis, although CLEC-2 maintains vascular integrity at sites of inflammation in the skin. There is accumulating evidence that the CLEC-2-podoplanin interaction plays an important role in thromboinflammation due to the upregulation of podoplanin on tissue-resident macrophages and stromal cells (57). The exact role of CLEC-2 in arterial thrombosis is not completely clear. However, in a mouse model of deep vein thrombosis, comprising inflammatory events, CLEC-2 deficient mice or mice treated with an anti-podoplanin antibody demonstrated substantially decreased thrombus formation (60). In addition, podoplanin can be highly expressed on tumor cells and the platelet CLEC-2/podoplanin axis was shown to promote tumor progression, metastasis, and cancer-induced thrombosis (61).

P-selectin, also known as CD62P, GMP-140, PADGEM (platelet activation-dependent granule external membrane protein), is a transmembrane single-chain glycoprotein (62) and the largest among the selectin family (63). Platelet P-selectin is embedded on the membrane of α-granules and also stored in Weibel-Palade bodies of vascular endothelial cells. Upon platelet activation, the membrane of α-granules merge with the platelet membrane *via* exocytosis, leading to P-selectin translocation to the platelet surface where it is rapidly cleaved off or slowly internalized, resulting in the release of soluble P-selectin (sP-selectin), whereas endothelial surface P-selectin is internalized within 30 min (21, 32, 64). The platelet surface P-selectin is usually referred to as CD62P and can be measured by flow cytometry in contrast to plasma released sP-selectin. The shedding mechanism remains unknown (21). It was shown that binding of platelet P-selectin to P-selectin glycoprotein ligand-1 (PSGL-1) on leukocytes leading to leukocyte rolling (65) and endothelial cells is required for P-selectin shedding, but the protease responsible for this is not discovered yet (66). Platelet-leukocyte aggregates (PLA) can be detected in blood and recognized as one of the most reliable markers for platelet activation (67). There is evidence from mice studies that rather dimeric than monomeric sP-selectin contributes to activation of leukocytes, thereby promoting vascular leukocyte recruitment and microvesicle formation (68).

Several studies acknowledged that the plasma level of sP-selectin originates predominantly from platelets, even though it may also be an indicator of endothelial cell activation, hence plasma levels of sP-selectin have been recognized as a biomarker of activated and degranulated platelets (64, 69, 70). This was also supported by the positive correlation between the level of sP-selectin and platelet count. SP-selectin activates leukocytes and promotes their adhesion to platelets (68).

Similar to P-selectin, CD40L (CD154 or GP39) is another externalized surface protein, which has potent pro-inflammatory properties (71) and belongs to the cytokine tumor necrosis factor (TNF) family (72). CD40L is detected on the surface of various cells including hematopoietic cells, like platelets, basophils, monocytes, macrophages, and non-hematopoietic cells such as mast, endothelial, and smooth muscle cells (72), suggesting a broad range of CD40L functions *in vivo* (73). Upon platelet activation by collagen or thrombin, CD40L, also located within the α-granule membrane, is mobilized to the platelet surface (21) and is enzymatically cleaved by MMP-2 and MMP-9 within a period of minutes to hours to generate soluble CD40L (sCD40L) (74). Despite the numerous sources of CD40L mentioned above, it was estimated that more than 95% of plasma sCD40L is derived from activated platelets and therefore might reflect the platelet activation status (75). SCD40L increases thrombus stability and promotes the expression of tissue factor, chemokines, and pro-inflammatory biological response modifier molecules (76).

**Figure 1:**
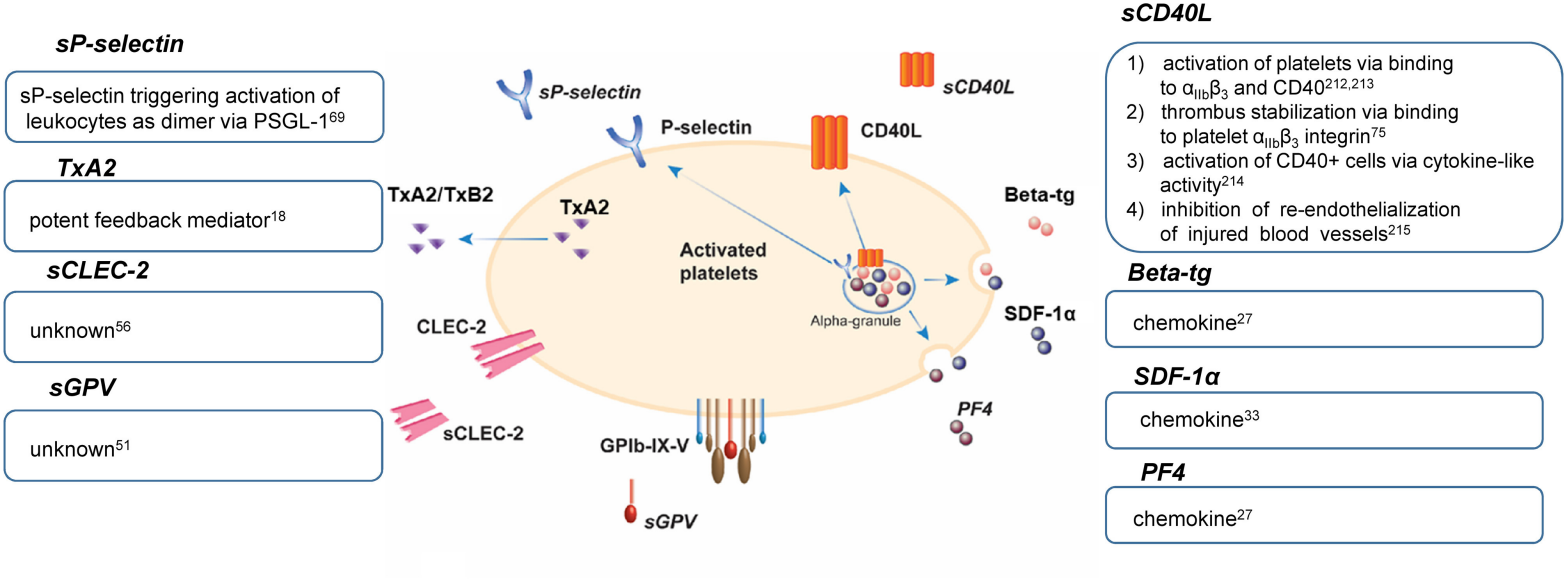
Physiological functions of platelet release factors (77–80). beta-tg, β-thromboglobulin; CD40L, CD40 ligand; CLEC-2, C-type lectin-like receptor 2; GP, glycoprotein; PF4, platelet factor 4; PSGL-1, P-selectin glycoprotein ligand-1; SDF-1α, stromal cell-derived factor-1α; TxA2, thromboxane A2; TxB2, thromboxane B2; s, soluble.

The shedding of receptor ectodomains represents an efficient mechanism for the irreversible downregulation of receptor expression on the platelet surface, resulting in decreased ligand binding. This leads to an essential and tight control of platelet responsiveness in primary hemostasis and coagulation but also in inflammatory processes where activated platelets modulate the activation state of leukocytes and vascular cells through direct receptor/glycoprotein-mediated interactions. The physiological functions of released factor from platelets are summarized in Figure 1.

## Platelet Biomarkers In Thrombotic Diseases

### Arterial Thrombosis

#### Coronary Artery Disease

Atherosclerosis is a systemic chronic disease resulting from lipid accumulation in the intima of arteries and chronic inflammation accompanied by platelet activation (81, 82). Coronary artery disease (CAD), defined by the presence of significant atherosclerosis within one or more major coronary arteries, is prone to trigger atherothrombosis on ruptured or eroded atherosclerotic plaques (83), a process in which platelets play a dominant role.

Several studies reported increased levels of platelet biomarkers in patients with CAD, demonstrated mainly by elevated levels of sP-selectin (84–87), sCD40L (86, 88), and sGPV (84, 86) compared to healthy individuals or non-CAD patients (Table 1). Lindmark et al. (85) reported elevated levels of platelet-monocyte (PMA) and platelet-neutrophil aggregates (PNA) measured by flow cytometry. The PMA and PNA levels were significantly higher in patients with unstable CAD vs. stable CAD, who in turn were characterized by slightly but not significantly higher levels compared to controls. Details of these studies are presented in Supplementary Table 1. The levels of sP-selectin were shown to be comparable between South Asian and white European CAD patients on antiplatelet drugs (89).

**Table 1:** Soluble biomarkers of platelet activation in coronary artery disease (CAD) patients.

**Biomarker**	**Clinical phenotype**	**Acute/chronic phase**	**Results: ↑, ↓, =**	**Study group**	**Reference group**	**References**
sP-sel	CAD	Chronic	↑	CAD patients on aspirin and dalteparin	HV	(84–86)
	CAD	Chronic	↑	CAD Patients	Hospitalized non-CAD patients	(87)
	CAD	Chronic	=	White Europeans with CAD on antiplatelet drugs	South Asians with CAD on antiplatelet drugs	(89)
	CAD	Chronic	↓	CAD patients on aspirin and heparin after clopidogrel administration	CAD patients on aspirin and heparin before clopidogrel administration	(90)
sCD40L	CAD	Chronic	↑	CAD patients on antiplatelet drugs	HV	(86)
	CAD	Chronic	↑	CAD patients with MI on aspirin	Hospitalized CAD patients without MI and non-CAD patients	(88)
	CAD	Chronic	=	CAD patients on aspirin and clopidogrel with: ACS (myocardial infarction or unstable angina), non-fatal ischemic stroke or transient ischemic attack, cardiovascular death, hospitalization for revascularization	Hospitalized CAD patients on aspirin and clopidogrel without ischemic events	(91)
	CAD	Chronic	↓	CAD patients on aspirin and clopidogrel	CAD patients on aspirin alone	(91, 92)
sCD40L	CAD	Chronic	↓	CAD patients on aspirin and heparin after clopidogrel administration	CAD patients on aspirin and heparin before clopidogrel administration	(90)
beta-tg	CAD	Chronic	↑	CAD patients with high on-aspirin RPR	CAD patients with low on-aspirin RPR	(93)
sGPV	CAD	Chronic	↑	CAD patients on antiplatelet drugs	HV	(84, 86)
SDF-1α	CAD	Chronic and acute	↑	CAD patients on aspirin with a stroke or dead	CAD patients without the primary endpoints	(94)

*Beta-tg, β-thromboglobulin; CAD, coronary artery disease; HV, healthy volunteers; MI, myocardial infarction; RPR, residual platelet reactivity; sCD40L, soluble CD40 ligand; SDF-1α, stromal cell-derived factor 1; sGP, soluble glycoprotein; sP-sel, soluble P-selectin. ↑, Increased compared to healthy controls; ↓, Decreased compared to healthy controls; =, Unchanged compared to healthy controls*.

Various research groups have suggested that the administration of antiplatelet drugs leads to a decline in the level of platelet activation biomarkers. The level of sP-selectin was lower in patients with stable CAD receiving aspirin compared to aspirin-naïve patients (90), while 5-day aspirin administration did not influence the level of sP-selectin in another study (84). The association between on-aspirin platelet reactivity and the level of beta-tg was studied by Pettersen et al. (93) who demonstrated that CAD patients with high residual platelet reactivity (RPR) had a higher level of beta-tg. At the same time, these patients were not characterized by hypercoagulability based on thrombin generation, and hence the authors speculated that the high on-aspirin RPR would rather depend on increased endothelial cell and platelet activation. However, no clinical outcomes were investigated for further exploration.

There is evidence that clopidogrel administration to aspirin-treated patients with CAD significantly reduced the levels of sP-selectin and sCD40L (90–92). In the study of Kaufman et al. (90), the decrease in sP-selectin and sCD40L after administration of a loading dose of clopidogrel did not correlate with platelet reactivity, indicating that the decline in soluble protein levels was likely due to initially elevated levels as a consequence of the percutaneous coronary intervention (PCI) procedure. The level of sP-selectin correlated moderately with sCD40L levels and platelet aggregation in response to arachidonic acid, ADP, and collagen, revealing a link between platelet activity and platelet aggregability (90).

The elevation of sCD40L is particularly evident in patients with recent MI who had higher levels of sCD40L than patients with non-MI CAD, or no CAD-patients (88). Higher sCD40L was accompanied by increased platelet activation as evidenced by increased PMA, PNA, and platelet-surface activated α_IIb_β_3_, determined by flow cytometry in whole blood. The extent of platelet activation was related to CAD stability, with the highest platelet activation in recent-MI patients. However, platelet CD62P did not differ between the groups. SCD40L was associated with female gender, hematocrit, and C-reactive protein and inversely associated with hypertension (91). However, no associations between sCD40L and clinical outcomes were noted in this study.

SGPV levels have been found increased in CAD patients (84, 86), one study appraised sGPV as a relevant biomarker for atherosclerotic patients (84). The second study indicated that platelet activation probably better correlates with intima-media thickness than with angiographic severity of CAD or may reflect thrombogenic abnormalities (86).

Ghasemzadeh et al. (94) reported in a study population of 599 patients that higher plasma SDF-1α level was associated with a nearly 5- and 6-fold increase in the risk of MI and cardiovascular death, respectively, providing a potentially powerful prognostic tool for patients with CAD.

#### Peripheral Artery Disease

Peripheral artery disease (PAD) is a severe systemic manifestation of atherosclerosis that typically becomes symptomatic in the legs (claudication) but also carries a high risk for MI and ischemic stroke. Similar to symptomatic CAD, patients with confirmed PAD who are treated with antiplatelet and possible anticoagulant drugs, are characterized by increased levels of sP-selectin (95–99), sCD40L (98), and sGPV (84) compared to healthy volunteers (Table 2, Supplementary Table 1).

**Table 2:** Soluble biomarkers of platelet activation in peripheral artery disease (PAD) and atherosclerotic patients.

**Biomarker**	**Clinical phenotype**	**Acute/chronic phase**	**Results: ↑, ↓, =**	**Study group**	**Control group**	**References**
sP-sel	PAD	Chronic	↑	PAD patients on aspirin, clopidogrel, warfarin	HV	(95–99)
	PAD	Chronic	↑	PAD patients on aspirin and vitamin K-antagonists with restenosis	PAD patients on aspirin and vitamin K-antagonists without restenosis	(100)
	Ischemia, PAD	Chronic	↑	Chronic limb ischemia on aspirin	Non-PAD controls on aspirin	(101)
	Ischemia, PAD	Chronic	↑	Chronic limb ischemia, PAD patients on aspirin	PAD patients on aspirin	(101)
	PAD	Chronic	=	PAD patients on aspirin	Non-PAD controls on aspirin	(101)
	PAD	Chronic	=	PAD patients on no drugs	HV	(102)
	PAD	Chronic	=	PAD patients on aspirin and clopidogrel	PAD patients on aspirin	(103)
	Atherosclerotic disease in type 2 diabetes	Chronic	↑	Type 2 diabetic patients with atherosclerotic disease on aspirin	HV and type 2 diabetic patients without atherosclerotic disease	(104)
sCD40L	PAD	Chronic	↑	PAD patients on aspirin, clopidogrel, and warfarin	HV	(98)
	PAD	Chronic	=	PAD patients on aspirin and vitamin K-antagonists with restenosis	PAD patients on aspirin and vitamin K-antagonists without restenosis	(100)
sCD40L	PAD	Chronic	=	PAD patients on aspirin and clopidogrel	PAD patients on aspirin alone	(103)
	Atherosclerotic disease in type 2 diabetes	Chronic	↑	Type 2 diabetic patients with and without atherosclerotic disease on aspirin	HV	(104)
PF4	Ischemia, PAD	Chronic	↑	Chronic limb ischemia on aspirin	Non-PAD controls on aspirin	(101)
	Ischemia, PAD	Chronic	=	Chronic limb ischemia, PAD patients on aspirin	PAD patients on aspirin	(101)
	PAD	Chronic	=	PAD patients on aspirin	Non-PAD controls on aspirin	(101)
sGPV	Coronary and Peripheral Atherosclerosis	Chronic	↑	Patients with coronary and peripheral atherosclerosis on aspirin	HV	(84)
11-DH-TXB2	PAD, presumed	Chronic	↑	Patients with presumed PAD on no drugs	HV	(102)

*11-DH-TXB2, 11-dehydrothromboxane B2; HV, healthy volunteers; PAD, peripheral artery disease; PF4, platelet factor 4; sCD40L, soluble CD40 ligand; sGP, soluble glycoprotein; sP-sel, soluble P-selectin; TSP-1, thrombospondin-1. ↑, Increased compared to healthy controls; ↓, Decreased compared to healthy controls; =, Unchanged compared to healthy controls*.

The level of sP-selectin was also higher compared to healthy volunteers in a study that included patients with presumed PAD, as well as those in whom the diagnosis was confirmed (102). SP-selectin correlated with the severity of PAD (99). This is also confirmed by Zamzam et al. (101), who demonstrated sP-selectin and PF4 were significantly higher in a group with chronic limb-threatening ischemia compared with non-PAD controls but did not differ between PAD and non-PAD groups. The levels of sP-selectin were significantly higher in type 2 diabetic patients with the atherosclerotic disease compared to patients with type 2 diabetes only or healthy subjects, while sCD40L levels were significantly elevated in diabetes patients compared to control subjects, with no difference between two diabetic subgroups (104).

Platelet surface CD62P and CD63 (a dense granule and lysosome membrane glycoprotein), as well as sP-selectin, were higher in the patients compared to the control group (98). CD62P well (*r* = 0.525) and mildly (*r* = 0.314) but significantly correlated with CD63 and sP-selectin, respectively. Tsakiris found no correlation between CD62P and sP-selectin (100). SCD40L failed to correlate with any of platelet activation markers both in the study of Blann et al. (98) and Tan et al. (104).

Tsakiris et al. (100) investigated the association between sP-selectin and sCD40L levels in relation to the development of restenosis within 6 months after peripheral angioplasty in patients with PAD. While sP-selectin was associated with outcome (restenosis), no such association was found for sCD40L. SCD40L was suggested to be more linked to endothelial activation due to its correlation with other endothelial activation markers (100). Eikelboom et al. (103) addressed the additive effect of clopidogrel-mediated platelet inhibition on top of aspirin treatment, showing inhibition of ADP- and collagen-induced platelet aggregation, but no reduction in sP-selectin and sCD40L.

Since sCD40L did not correlate with any other markers of platelet activation (sP-selectin, CD62P, CD63) (98, 104), sP-selectin may be a more reliable biomarker for atherosclerotic patient stratification than sCD40L. Burdess et al. (105) criticized the use of sP-selectin and sCD40L measured by ELISA due to the lack of consistency of measured levels in the same group of patients within 1 day and between days and found poor and no correlation with flow cytometry results confirming results of Tsakiris et al. (100), Blann et al. (98), and Tan et al. (104).

Platelet biomarkers in relation to atherosclerotic risk factors were addressed in a population-based study with nearly 3,000 participants; no significant associations were found between sCD40L level and the risk factors (106). The authors also claimed that sCD40L is not a useful tool to screen for subclinical atherosclerosis. Another study included more than 300 patients with atherosclerosis after PCI with endovascular stent implantation (107) and found a strong correlation of PF4, TSP-1, and sCD40L with each other as well as with peak thrombin generation and endogenous thrombin potential, while sP-selectin only correlated weakly with TSP-1. This was explained by assuming that PF4, TSP-1, and sCD40L are mostly of platelet origin, whereas sP-selectin is primarily released by endothelial cells in patients with advanced atherosclerosis.

SGPV was increased in both PAD and CAD patients compared to healthy subjects but was insensitive to 5 days of aspirin treatment (84). Since we found only two studies that measured sGPV in PAD and CAD patients (84, 86), current data on this biomarker are still limited.

#### Acute Coronary Syndrome and Myocardial Infarction

Acute coronary syndrome (ACS) describes the predominant situation of symptomatic CAD due to ischemia of the heart, oftentimes in response to atherothrombotic occlusion (108). CAD is the most common cause of arterial thrombosis in ACS and MI. Platelets are the main culprits in the development of ACS and subsequent cardiovascular events (109).

While many studies, as discussed above, show evidence of increased platelet activity in CAD, studies in ACS focus more on the dynamics of platelet release markers in response to pharmacological intervention. Studies investigating the effect of α_IIb_β_3_ antagonists on the release of platelet biomarkers showed reduced sCD40L release from activated platelets (110, 111) (Table 3, Supplementary Table 1). In the study by Ray et al. (111), sCD40L was associated with coronary thrombosis and three different treatments were compared: bivalirudin alone, bivalirudin with α_IIb_β_3_ inhibitors, and unfractionated heparin (UFH) with α_IIb_β_3_ inhibitors. Bivalirudin, the direct thrombin inhibitor, in large-scale randomized trials has been demonstrated to reduce bleeding and thrombocytopenia compared to heparin plus α_IIb_β_3_ inhibitors, while ischemia rates in patients after PCI were similar (112). Ray et al. (111) described levels of sCD40L to be significantly lower in the UFH group compared with the other two groups, indicating that UFH combined with α_IIb_β_3_ inhibitors reduced sCD40L release more strongly than bivalirudin with or without α_IIb_β_3_ inhibitors.

**Table 3:** Soluble biomarkers of platelet activation in acute coronary syndrome (ACS) and acute myocardial infarction (AMI) patients.

**Biomarker**	**Clinical phenotype**	**Acute/chronic phase**	**Results: ↑, ↓, =**	**Study group**	**Reference group**	**References**
sCD40L	ACS	Chronic	↓	ACS patients on aspirin, clopidogrel, and abciximab or eptifibatide (α_IIb_β_3_ antagonists)	ACS patients on aspirin, clopidogrel, and no α_IIb_β_3_ antagonists	(110)
	ACS	Acute	↑	ACS patients on bivalirudin and provisional α_IIb_β_3_ inhibition	ACS patients on UFH and mandatory α_IIb_β_3_ inhibitors	(111)
	ACS	Chronic and acute	↑	ACS patients with “high” platelet aggregability on aspirin, clopidogrel, thienopyridine, ticlopidine, enoxaparin	ACS patients with “low” platelet aggregability on aspirin, clopidogrel, thienopyridine, ticlopidine, enoxaparin	(113)
sGPVI	ACS	Acute	↑	ACS patients with high platelet count	ACS patients with low platelet count	(109)
sP-sel	AMI	Chronic	↑	AMI patients on antiplatelet therapy	HV	(114)
	AMI	Chronic	↑	AMI patients on aspirin, ticagrelor, and heparin	Stable angina patients on antiplatelet therapy	(115)
	AMI	Chronic	↑	AMI patients on aspirin, ticagrelor, and heparin	Outpatients without history of coronary heart diseases on antiplatelet therapy	(115)
	AMI	Chronic	=	AMI patients after 3–6 months of recovery on aspirin	Acute MI patients on aspirin	(116)
	AMI	Acute	↓	AMI patients on dual therapy with ximelagatran and aspirin	AMI patients on aspirin only	(117)
sCD40L	AMI	Acute	↑	AMI patients on aspirin and clopidogrel	HV	(118)
	AMI	Acute and chronic	↑	AMI patients on aspirin	Stable CAD patients on aspirin	(119)
	AMI	Acute	=	AMI patients on aspirin and statins	AMI patients on aspirin only	(120)
sCD40L	Post-MI in diabetes mellitus	Chronic	=	AMI patients with DM and non-DM on aspirin, abciximab, eptifibatide, and heparin	HV	(121)
	AMI	Acute	↓	AMI patients with thrombectomy	AMI patients without thrombectomy	(122)
sGPV	AMI	Acute	↑	AMI patients on aspirin, abciximab, and heparin	HV	(123)
	AMI	Acute	↑	AMI patients with DM and non-DM on aspirin, abciximab, eptifibatide, and heparin	HV	(121)
	AMI	Acute	↑	AMI patients on aspirin, clopidogrel, abcixibam, eptifibatide, ticlopidine, and heparin	HV	(124)

*ACI, acute cerebral ischemia; ACS, acute coronary syndrome; AMI, acute myocardial infarction; CAD, coronary artery disease; HDL, high-density lipoprotein; HV, healthy volunteers; sCD40L, soluble CD40 ligand; SDF-1α, stromal cell-derived factor 1; sGP, soluble glycoprotein; sP-sel, soluble P-selectin; UFH, unfractioned heparin; DM, diabetes mellitus. ↑, Increased compared to healthy controls; ↓, Decreased compared to healthy controls; =, Unchanged compared to healthy controls*.

The second study described the reducing effect of treatment with two different α_IIb_β_3_ antagonists (eptifibatide or abciximab) on plasma sCD40L levels after PCI compared to pre-PCI (110). This reduction was not observed in patients without α_IIb_β_3_ antagonists (control); baseline levels were comparable between the different treatment groups. For control patients not treated with clopidogrel before the PCI, clopidogrel administration at the end of the procedure reduced plasma sCD40L significantly 18–24 h after PCI. PMA followed a similar pattern, however, the correlation between the two markers was not assessed.

In another study, ADP-induced platelet aggregation was measured in ACS patients and the patients were subsequently divided into the “high aggregation” (above median) or “low aggregation” (below median) group (113). Elevated sCD40L and sP-selectin levels were found in ACS patients with relatively high platelet aggregability in response to ADP. The authors speculated that CD40L-related enhancement of inflammation and coagulation theoretically might increase the risk of restenosis and in-stent thrombosis in CAD patients and, as a prove, referred to several studies that found associations between restenosis and the CD40 system (125–127).

The study in ACS patients addressing sGPVI demonstrated an inverse correlation between plasma sGPVI levels and platelet count; comparable results were found for platelet surface-expressed GPVI levels. This suggests that patients with lower platelet counts have a higher platelet activation state and, in line with this, these patients were prone to have poorer clinical outcomes (composite of MI, stroke, cardiovascular death) (109). So far, only one study addressed sGPVI levels in relation to platelet count in ACS patients (109). Other correlations with the severity of the disease or clinical outcomes were not described.

In patients with acute MI (AMI) there is evidence for elevated plasma levels of sP-selectin (128), sCD40L (118), and sGPV (121, 123, 124) compared to healthy individuals. A constant elevation of sP-selectin in patients with AMI over the period of 3–6 months was presented by Christersson et al. (117) and Järemo et al. (116). Patients with AMI had significantly higher sP-selectin levels compared with stable angina patients and outpatients without a history of coronary heart disease (115). In the study of Christersson et al. (117), aspirin-treated patients randomized to higher doses of a direct thrombin inhibitor showed lower sP-selectin levels than patients on lower doses or placebo. Another study reported no difference in the sP-selectin level in AMI patients at 3–6 h after infarction compared to 1 day after infarction (116). In the study of Huisse et al. (123), elevated plasma levels of sGPV were found in combination with increased flow cytometry assessed CD62P and activated α_IIb_β_3_ presentation *ex vivo* in AMI patients compared to healthy volunteers, confirming platelet activation by different tests.

Following up on the topic of drug-related effects on platelet release factors, the addition of statins to conventional aspirin therapy was not beneficial in AMI patients (120). Several articles addressed sCD40L in AMI patients in comparison to other thrombotic diseases. An elevated sCD40L level was observed in patients with AMI compared to age/sex-matched controls with stable CAD (119). In another study, sCD40L levels were higher in patients with MI and diabetes than in subjects with MI alone or those with diabetes alone. However, the difference was not statistically significant which might be explained by the low number of study subjects in each group (121). Interestingly, the levels of sCD40L distinguished not only patients with thrombus formation vs. control subjects but also patients with high-burden thrombus formation in the infarct-related artery vs. low-burden thrombus formation (118).

Together with sP-selectin and sCD40L, sGPV was also elevated in AMI and ACS patients (121, 124) and was recognized as a more sensitive marker of thrombus-induced platelet activation than platelet-derived microparticles (123). Similar to CAD, SDF-1α was also studied in AMI patients, showing that increased SDF-1α levels were associated with the risk factors older age, lower levels of high-density lipoprotein (HDL) cholesterol, and smoking. After adjustment for these factors, SDF-1α correlated with incident heart failure and all-cause mortality (129).

#### Acute Ischemic Stroke

Ischemic stroke, not based on cardiac embolism, is predominantly a consequence of atherothrombosis in the carotid and other cranial arteries (130). Platelet activation as measured by release markers during an acute ischemic stroke (AIS) has been demonstrated in many studies, showing elevated plasma levels of sP-selectin (131–138), sCD40L (134), sGPVI (139), and sCLEC2 (140, 141) in comparison to healthy volunteers (Table 4, Supplementary Table 1).

**Table 4:** Soluble biomarkers of platelet activation in acute ischemic stroke (AIS) patients.

**Biomarker**	**Clinical phenotype**	**Acute/chronic phase**	**Results: ↑, ↓, =**	**Study group**	**Control group**	**References**
sP-sel	AIS	Chronic	↑	ACI patients on no drugs, aspirin, clopidogrel, ticlopidin or warfarin	HV	(131)
	AIS	Acute	↑	ACI patients on either aspirin or clopidogrel	HV	(132)
	AIS	Acute	↑	AIS patients on no drugs	HV	(133)
	AIS	Acute	↑	AIS patients on aspirin	HV	(134, 135)
	AIS	Acute	↑	AIS patients	HV	(136, 137)
	Cerebral ischemic event and/or carotid stenosis	Chronic	↓	Patients on 10-day treatment with terutroban or aspirin plus clopidogrel	Patients on aspirin (day 0)	(142)
sCD40L	AIS	Acute	↑	AIS patients on aspirin	HV	(134)
	AIS	Acute	=	AIS patients on aspirin and warfarin	Individuals without coronary atherosclerosis	(143)
PF4	AIS	Chronic	↑	AIS patients on no drugs, aspirin, clopidogrel, heparin or warfarin	Hospitalized patients with a chronic non-vascular neurological disorder	(144)
sGPV	AIS	Chronic	↑	AIS patients on no drugs, aspirin, clopidogrel, heparin or warfarin	Hospitalized patients with a chronic non-vascular neurological disorder	(144)
sGPVI	AIS	Acute	↑	AIS patients	HV	(139)
	AIS	Chronic	↓	AIS patients on aspirin, clopidogrel, or vitamin K antagonists	Hospitalized patients without AIS	(145)
sCLEC2	AIS	Acute	↑	AIS patients	HV	(140, 141)

*AIS, acute ischemic stroke; HV, healthy volunteers; PF4, platelet factor 4; sCD40L, soluble CD40 ligand; sCLEC-2, soluble C-type lectin-like receptor 2; sGP, soluble glycoprotein; sP-sel, soluble P-selectin; ACI, acute cerebral ischemia. ↑, Increased compared to healthy controls; ↓, Decreased compared to healthy controls; =, Unchanged compared to healthy controls*.

In several studies, sP-selectin again was an indicator of platelet activation and increased levels were found in patients with AIS, independent of treatment with antithrombotics (133–137). This increase was also reported for CD62P (134). In a randomized study with patients at high risk of recurrent IS, treatment groups with different antiplatelet therapies (APT) were compared. A significant reduction in sP-selectin was demonstrated 10 days after treatment with terutroban or clopidogrel plus aspirin, while a decreasing trend was reported after treatment with aspirin or terutroban plus aspirin (142). Spontaneous and arachidonic acid-induced platelet aggregation was either low or decreased both at baseline (day 0) and day 10. The impaired aggregation response is an expected observation since patients were on aspirin during the run-in period. In that case, sP-selectin might be recognized as a more sensitive marker for assessing the antiplatelet drug effect. Both aspirin and clopidogrel lowered the sP-selectin level in patients with acute cerebral infarction (132). SP-selectin in this study positively correlated with flow cytometry detected PMA (*r* = 0.454, *P* < 0.05). Additionally, the prognostic value of sP-selectin levels was underlined by its strong correlation with the onset time of progressive IS (137).

In one study, plasma levels of sCD40L and platelet CD62P were found to be similar in AIS patients compared to controls (143). However, platelet surface CD40L expression and PMA levels were higher in patients compared to controls as assessed with flow cytometry. The control group included individuals without coronary atherosclerosis but with similar treatment and risk factors for cardiovascular diseases, which might explain the lack of difference in the level of sCD40L. The lack of significance might also be due to the small sample size of 41 patients vs. 10 controls.

SGPV was elevated in patients with AIS compared to control patients without vascular complications and antithrombotic treatment; this sGPV increase was not influenced by antithrombotic treatment (144). Multivariate analysis demonstrated a correlation between sGPV and stroke, platelet, and leukocyte counts, but not with cardiovascular risk factors. Interestingly, sGPV positively correlated with the PF4 level.

There are two studies where sGPVI was measured; elevated sGPVI levels were found in IS patients compared to healthy volunteers (139), while reduced sGPVI levels were seen in comparison to patients with non-ischemic events (145). In the latter study, the control group consisted of patients with other cerebral disorders, which might distort the interpretation of the sGPVI level in IS patients. Additionally, Wurster et al. (145) evaluated GPVI levels in chronic IS patients whereas Al-Tamimi et al. (139) investigated acute phase patients. Interestingly, Wurster et al. (145) did report increased levels of platelet-surface GPVI in IS patients. Inconsistency between soluble and platelet-surface expressed GPVI levels might be explained by the method used to measure sGPVI, since a newly developed ELISA assay was applied.

Two articles originating from the same cohort of AIS patients consisting of 323 individuals with a follow-up of 1 year showed that sCLEC-2 might be used as a predictor for AIS; the elevated level of the biomarker was significantly correlated with stroke progression and death. Patients with the highest sCLEC-2 level had an 8-fold higher risk of progressive stroke or death compared to the patients in the lowest quartile (140, 141).

### Atrial Fibrillation

Although atrial fibrillation (AF) is currently considered a condition that in the vast majority of cases requires oral anticoagulation to prevent thromboembolic stroke, research from past decades also considered the role of platelets in this setting (146–148). For this reason, there is quite some literature on the involvement of activated platelets in AF-related hypercoagulability. Many studies reported elevated levels of sP-selectin (149–156) and sCD40L (154, 157, 158) in patients with AF compared to healthy subjects. In addition, increased concentrations of plasma beta-tg (84, 151, 159, 160) and sGPV (160) were documented (Table 5, Supplementary Table 1). Choudhury et al. (153) additionally measured platelet surface CD62P and CD63 by flow cytometry in whole blood. Both markers were elevated in AF patients as well as sP-selectin compared to healthy people. However, CD62P strongly correlated with CD63 (r = 0.6; *p* < 0.001), but not with sP-selectin.

**Table 5:** Soluble biomarkers of platelet activation in atrial fibrillation (AF) patients.

**Biomarker**	**Clinical phenotype**	**Acute/chronic phase**	**Results: ↑, ↓, =**	**Study group**	**Control group**	**References**
sP-sel	AF	Chronic	↑	AF patients on no drugs	HV	(149, 150)
	AF	Chronic	↑	AF patients on aspirin, clopidogrel, and warfarin	HV	(151–156)
	AF	Chronic	=	AF patients on aspirin and warfarin	HV	(70, 159, 161–167)
	AF	Chronic	=	AF patients on aspirin and warfarin	AF patients on warfarin only	(70)
	AF	Chronic	=	AF patients with vascular events on aspirin	AF patients without vascular events on aspirin	(162)
	AF	Chronic	=	AF patients with worse renal function	AF patients with better renal function	(168)
	AF	Chronic	=	AF patients with atrial thrombus on antiplatelet and anticoagulant drugs	AF patients without atrial thrombus on antiplatelet and anticoagulant drugs	(169)
	AF	Chronic	=	AF patients with hypertension on aspirin and warfarin	AF patients with normotension on aspirin and warfarin	(170)
	AF	Chronic	=	AF patients on apixaban	AF patients on rivaroxaban	(171)
	AF	Chronic	↓	AF patients on no drugs	HV	(172)
sCD40L	AF	Chronic	↑	AF patients on aspirin, clopidogrel, and warfarin	HV	(154, 157, 158)
	AF	Chronic	↑	AF patients with thrombotic events on warfarin	AF patients without thrombotic events on warfarin	(173)
	AF	Chronic	↑	AF patients with atrial thrombus on antiplatelet and anticoagulant drugs	AF patients without atrial thrombus on antiplatelet and anticoagulant drugs	(169)
	AF	Chronic	↑	AF patients with stroke and MI on aspirin and anticoagulants	AF patients without stroke and MI on aspirin and anticoagulants	(174)
beta-tg	AF	Chronic	↑	AF patients on aspirin, clopidogrel, and warfarin	HV	(84, 151, 159, 160)
	AF	Chronic	=	AF patients on no drugs	HV	(164)
	AF	Chronic	=	AF patients on no warfarin	HV	(172)
	AF	Chronic	=	AF patients on apixaban	AF patients on rivaroxaban	(171)
sGPV	AF	Chronic	↑	AF patients on aspirin and warfarin	HV	(160)
sGPVI	AF	Chronic	↓	AF patients on apixaban or rivaroxaban	AF patients on warfarin	(175)
TSP-1	AF	Chronic	=	AF patients on apixaban	AF patients on rivaroxaban	(171)

*AF, atrial fibrillation; beta-tg, β-thromboglobulin; HV, healthy volunteers; MI, myocardial infarction; sCD40L, soluble CD40 ligand; sGP, soluble glycoprotein; sP-sel, soluble P-selectin; TSP-1, Thrombospondin-1. ↑, Increased compared to healthy controls; ↓, Decreased compared to healthy controls; =, Unchanged compared to healthy controls*.

However, some studies found no difference or even a decrease in biomarker levels when comparing patients with AF and controls. This was observed most strikingly for sP-selectin. Yet the majority of articles demonstrating no difference between the groups adjusted the association between sP-selectin levels and AF severity or prognosis for confounding factors. For example, sP-selectin in AF patients correlated with diabetes but not with other recognized AF risk factors such as increasing age, recent heart failure, and prior cerebral ischemia (70). The absence of an association between sP-selectin levels and AF or cardiovascular risk was again claimed by this group a year later (161, 162). In one of their studies, Conway et al. (70) pointed out that the lack or absence of adequate adjustment for cardiovascular diseases may falsely link changes in sP-selectin levels to AF.

The level of sP-selectin was found to be unrelated to clinical outcomes (IS, MI, or vascular death) (162) and left atrial thrombus formation (169) in AF patients. Similar sP-selectin levels were reported when comparing different treatment groups; patients on warfarin plus aspirin vs. warfarin alone (70) or patients on rivaroxaban vs. apixaban (171). In the study by Steppich et al., rivaroxaban and apixaban did not influence levels of beta-tg and TSP-1. However, these direct oral anticoagulants were found to be more effective than warfarin in suppressing sGPVI measurements (175).

SCD40L was elevated in AF patients with embolic events, atrial thrombus formation (169, 173), stroke, and MI (174) compared to AF patients without these conditions. Other studies provide evidence that sCD40L is inversely related to stroke risk (176). In one of the largest studies including 880 subjects, Lip et al. reported that patients at the highest risk of stroke as determined by increased age and blood pressure, impaired left ventricular function, and previous thromboembolism, had lower levels of sCD40L than people without any of these factors. SCD40L, in contrast to sP-selectin and beta-tg, was a prognostic biomarker for vascular events in AF patients (173, 174). No correlation was found between sCD40L and sP-selectin in the study of Choudhury et al. (154).

Beta-tg levels were higher in patients with AF and similar to sP-selectin indifferent to aspirin (159, 160), warfarin (160, 172), rivaroxaban, and apixaban (171) administration. No relation was found between platelet aggregation induced by ADP, collagen, epinephrine, and thrombin and the plasma platelet activation markers sP-selectin (151), beta-tg (151, 160), and sGPV (160).

One of the unsolved questions in AF research is whether any of the observed changes in platelet biomarkers reflect the arrhythmia *per se*, or the comorbidity (152–154). An effect of AF was postulated based on two studies showing that lone AF was associated with elevated sP-selectin compared to age-matched controls. Lone AF patients had also enhanced sGPV levels (160) further supporting a role of platelet activation in AF since sGPV comes exclusively from platelets.

### Venous Thrombosis

Despite the fact that venous thrombosis is traditionally not regarded a condition that is dependent on platelet activation, clinical studies have clearly shown a protective effect of low-dose aspirin on recurrent venous thromboembolism (VTE). This effect is most likely explained by the inhibition of platelets as the low dose of aspirin does not have any demonstrable anti-inflammatory effects in humans (185). Although the effect of oral anticoagulation is clinically more relevant than APT to prevent recurrent VTE, the involvement of platelets in venous thrombosis remains of interest, particularly for settings in which the addition of APT may be considered, like in acute VTE or periprocedural, in case of venous stenting.

One of the most thorough explorations on platelet biomarkers in VTE was done by Riedl et al. (23), who studied several biomarkers and their mutual associations. The researchers compared sP-selectin, sCD40L, PF4, and TSP-1 among three groups: cancer patients with VTE, cancer patients without VTE, and healthy subjects. Interestingly, only sP-selectin was elevated among all biomarkers in cancer patients with VTE, compared to the other groups which were not different from each other, indicating that VTE rather than cancer was responsible for the sP-selectin increase (Table 6, Supplementary Table 1). Although TSP-1 was increased in both cancer groups compared to healthy volunteers, it was not affected by the presence of VTE. The authors concluded that sCD40L, PF4, and TSP-1 cannot predict VTE development, while sP-selectin, on the contrary, could have predictive potential (23). SCD40L, PF4, and TSP-1 mutually correlated with each other and weakly with sP-selectin which is released not only by platelets but also by endothelial cells. Based on this, Riedl et al. (23) suggested that VTE is more associated with endothelial rather than platelet activation.

**Table 6:** Soluble biomarkers of platelet activation in patients with venous thromboembolism (VTE).

**Biomarker**	**Clinical phenotype**	**Acute/chronic phase**	**Results: ↑, ↓, =**	**Study group**	**Control group**	**References**
sP-sel	DVT	Chronic	↑	DVT patients on anticoagulants	HV	(177–180)
PF4	DVT	Chronic	↑	DVT patients on anticoagulants	HV	(177)
sP-sel	VTE	Acute	↑	Patients with cancer-associated VTE	HV, cancer patients without VTE	(23)
	VTE	Chronic	↑	VTE patients with and without anticoagulant therapy	Subjects without VTE	(181)
	VTE	Acute	↑	VTE patients on no drugs with a recurrent event	VTE patients on no drugs without recurrent events	(182)
sCD40L	VTE	Acute	=	Patients with cancer-associated VTE	HV, cancer patients without VTE	(23)
	VTE	Chronic	=	VTE patients with and without anticoagulant therapy	Subjects without VTE	(181)
PF4	VTE	Acute	=	Patients with cancer-associated VTE	Cancer patients without VTE	(23)
TSP-1	VTE	Acute	=	Cancer patients with and without VTE	HV	(23)
TxB2	VTE	Acute	↑	VTE patients on aspirin, clopidogrel, vitamin K antagonists, heparin group, and direct FXa inhibitors	Subjects with excluded VTE on aspirin, clopidogrel, vitamin K antagonists, heparin group, and direct FXa inhibitors	(183)
sP-sel	Acute pulmonary embolism	Acute	↑	Acute pulmonary embolism patients on enoxaparin, heparin, and warfarin	HV	(184)

*DVT, deep vein thrombosis; HV, healthy volunteers; PF4, platelet factor 4; sCD40L, soluble CD40 ligand; sP-sel, soluble P-selectin; TSP-1, Thrombospondin-1; VTE, venous thromboembolism. ↑, Increased compared to healthy controls; ↓, Decreased compared to healthy controls; =, Unchanged compared to healthy controls*.

This conclusion is also supported by Migliacci et al. (181) who measured the level of sP-selectin and sCD40L in VTE patients and compared them to controls. However, it is important to mention that the control group included subjects with AF and valve prosthesis together with healthy volunteers. In accordance with the findings of Riedl et al., the level of sP-selectin was significantly higher in the patient group in contrast to sCD40L, which was similar between the patients and controls. In this study, also plasma vWF level was measured, which was higher in patients and correlated weakly but significantly with sP-selectin. The fact that vWF reflects endothelial activation and is stored together with sP-selectin in endothelial cells (186), supports the suggestion that endothelial activation is more pronounced in VTE patients compared to platelet activation and is responsible for the elevation of sP-selectin level in plasma.

In contrast to the above-mentioned study (23), Furio et al. (177) observed a significant increase in PF4 levels in deep vein thrombosis (DVT) patients compared to healthy volunteers. However, it should be considered that Riedl et al. (23) studied cancer patients with VTE in the acute phase, while Furio et al. (177) included patients with DVT in a chronic phase that, in addition to possible effects of anticancer treatment, may explain differences in results for this biomarker.

TxB2 was significantly elevated in patients with confirmed VTE diagnosis, independent of aspirin intake, in contrast to patients with excluded VTE (183). Non-aspirin VTE cases presented significantly shorter closure times with collagen/ADP and collagen/epinephrine in the platelet function analyzer compared to controls. Within the group of non-aspirin users, platelet aggregability in response to ADP or collagen was lower in VTE-cases compared to patients with excluded VTE. Patients with VTE showed higher platelet CD63 surface presentation *ex vivo* and lower platelet-dependent thrombin generation triggered by tissue factor, independent of therapy.

Other studies mostly concentrated on sP-selectin unanimously observing elevated levels of this biomarker in patients compared to healthy subjects, regardless of the location of venous thrombosis. SP-selectin was higher in patients with DVT (177–180) and acute pulmonary embolism (184). In line with elevated sP-selectin plasma levels in patients with DVT, Furio et al. (177) observed increased platelet CD62P presentation. In contrast, Chung et al. (184) reported unaltered platelet CD62P expression and PLA, but increased activated integrin α_IIb_β_3_ in patients with acute pulmonary embolism compared to controls. In the study of Kyrle et al. (182), sP-selectin appeared to be predictive for VTE recurrence, i.e., individuals with VTE and higher sP-selectin levels were more likely to have a second VTE event.

However, some studies questioned if sP-selectin reflects platelet and not endothelial activation since the levels of sP-selectin did not correlate with sCD40L (23, 181) and TSP-1 levels (23). Another study provided evidence that the elevated sP-selectin level and enhanced urinary 11-DH-TxB2 excretion in DVT patients was due to increased platelet activation (179). Therefore, we conclude that the question about the presence of platelet activation in DVT patients remains open. However, sP-selectin might be used as a prognostic tool for the recurrent VTE or incidence of VTE in cancer patients.

## Conclusions And Perspectives

Platelets are important contributors to the development of arterial thrombotic events. They are involved in atherosclerotic plaque formation and plaque rupture can lead to ischemia or infarction (187). Platelets are involved in thrombosis not only as the first violins of the blood coagulation process, but also as promoters of inflammation (188). The identification of changes in the level of plasma biomarkers associated with upcoming thromboembolic events could allow timely adjustment of the treatment strategy in order to prevent the disease aggravation. Therefore, biomarkers of platelet activation may become a valuable instrument for the prognosis of acute events.

An ideal biomarker should be specific, accurate, reproducible by a simple technique, independent from pre-analytical artifacts, cost-effective, and acceptable to patients (26, 32, 188). In contrast to plaque material, platelets are accessible through routine venipuncture and are easily counted within a minute by a standard cell counter. This allows serial sampling and long-term monitoring.

However, despite numerous clinical studies evaluating platelet biomarkers, data remain inconclusive. Several biomarkers were suggested but none of these can be recognized as a robust diagnostic marker. Interindividual variability and inconsistencies in cutoff values impede the implementation of biomarkers of platelet activation in the wide clinical practice. Besides this, the *ex vivo* manipulations like the blood drawing procedure and centrifugation can pre-activate platelets and distort real numbers of biomarkers level (189–192). The measurements can be also influenced by the type of anticoagulation, storage, and thawing procedures (32).

A common problem in the summarized clinical studies may be limited power due to small sample sizes and confounding due to incomplete or absent adjustment for risk factors for thrombosis, as identified as a problem in assessing sP-selectin levels in AF. There are many molecules expressed by platelets. Some of them are exclusive for platelets such as GPIbα, GPV, and GPVI, whereas others are also synthesized by other cells, e.g., P-selectin, CD40L, and SDF-1α. Our review demonstrates that the limelight of clinicians' attention was obviously mainly focused on sP-selectin and sCD40L. Currently, sP-selectin is recognized as an important marker of platelet activation and was found to be elevated in a broad range of conditions including various types of cardiovascular diseases (unstable angina, thrombocytopenia, arterial hypertension, stroke, AMI, congestive heart failure) as discussed, but also in other conditions, including autoimmune disorders (Sjogren's syndrome, systemic lupus erythematosus), diabetes, or psychiatric disorders (64, 193–200).

Increased values of sCD40L have been found in cardiovascular diseases including PAD (128), CAD (86), AF (154, 157, 158, 173, 174), AIS (134), and also in patients with diabetes (201). Similarly, beta-tg and PF4 were reported to be altered in other diseases too, including cancer (202–205), ischemic heart disease (206), and AF (207). SGPVI reflected activation of platelets in patients with AIS (139), microangiopathy (208), rheumatoid arthritis (59, 209), and Alzheimer's disease (210). Thus, it can be concluded that the above biomarkers do not specifically reflect thrombosis but probably also reflect diverse other processes some of which may in part be associated with platelet activation. At this stage, there are no data indicating distinct platelet activation profiles related to specific diseases, or predicting diseases (21).

Platelet activation is a well-known contributor to the pathogenesis of arterial thrombosis and leads to increased levels of platelet activation biomarkers. As discussed, some markers are platelet specific, whereas others may be increased due to activation of other cell types including endothelial cells. So far, it remains challenging to distinguish the exact input of platelet activation and vessel wall pathology into the increase of sP-selectin or sCD40L based on the data provided. Similarly, elevations of biomarkers and risk associations may vanish upon adjustment for confounding factors, as mentioned for AF.

**Figure 2:**
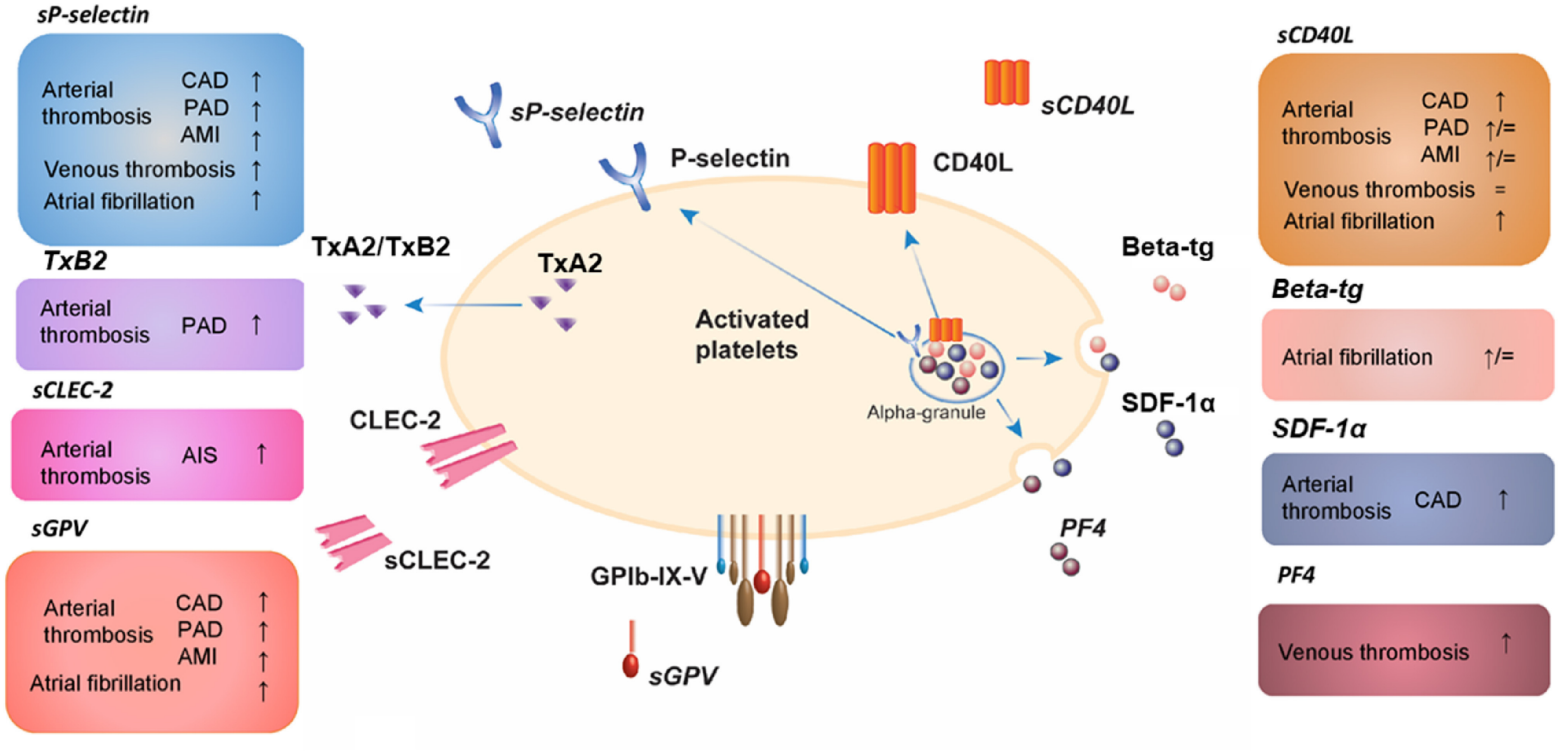
Platelet released activation biomarkers in arterial and venous cardiovascular diseases determined by ELISA-based tests. AIS, acute ischemic stroke; AMI, acute myocardial infarction; beta-tg, β-thromboglobulin; CAD, coronary artery disease; CD40L, CD40 ligand; CLEC-2, C-type lectin-like receptor 2; GP, glycoprotein; PF4, platelet factor 4; SDF-1α, stromal cell-derived factor-1α; TxA2, thromboxane A2; TxB2, thromboxane B2; PAD, peripheral artery disease; s, soluble; ↑, increased compared to healthy controls; ↓, decreased compared to healthy controls; =, unchanged compared to healthy controls.

Evidence for using platelet biomarkers as a prognostic and stratifying tool in DVT is still scarce. Interestingly, a recent study described platelet-related parameters in patients with confirmed VTE compared to patients with suspected but unconfirmed VTE, independent of the underlying cardiovascular profile (183). Herein VTE patients were characterized with elevated expression of platelet activation markers in combination with lower platelet-dependent thrombin generation *in vitro*. These findings clearly underscore the role of platelets in VTE. A current overview of platelet released activation biomarkers in arterial and venous cardiovascular diseases determined by ELISA-based tests is presented in Figure 2.

Several authors recommend implementing a combination of several biomarkers, which allows a more objective assessment of a patient's current state since the pathogenesis of thrombosis is a complex process involving the interplay between inflammation, coagulation, and cellular activation (211–214). It is also worthwhile to link biomarker assays to platelet function tests and platelet surface markers to obtain a more comprehensive understanding of the disease state.

It becomes obvious that there is a need for larger clinical trials to investigate the diagnostic potential of the biomarkers discussed in the thrombosis setting (32). The application of machine learning for the identification of signatures of platelet biomarkers could better reflect the biological complexity and multifactorial processes and overcome the high interindividual variability and limitations due to the scatter of measurement results. The newly available high-throughput protein technologies open up possibilities here that could lead to new insights.

Inclusion of newer, less well-studied plasma markers of platelet activation, such as sGPVI, sGPIbα, SDF-1α, sGPV, and sCLEC2, in clinical studies might be valuable in the search for reliable thrombotic biomarkers. For interpretation and comparison, future studies measuring biomarkers should ideally report detailed information on clinical parameters, pre-analytical and analytical variables. This information should be stratified and analyzed to determine its influence on the association between disease severity and biomarker level.

## Author Contributions

GB, MN, PM, and HC drafted the manuscript. All authors have seen and approved the final version of the manuscript, participated in the interpretation of the findings, reviewed the manuscript, and revised it critically before submission.

## Conflict of Interest

PW has received research funding outside the present study from Boehringer Ingelheim, Sanofi-Aventis, Bayer Healthcare, Daiichi Sankyo Europe, and Novartis, and received outside the present study honoraria for lectures or consulting from Boehringer Ingelheim, Bayer HealthCare, Evonik, AstraZeneca, and Sanofi-Aventis. The remaining authors declare that the research was conducted in the absence of any commercial or financial relationships that could be construed as a potential conflict of interest.

## Abbreviations

11-DH-TxB2: 11-dehydrothromboxane B2
ACS: Acute coronary syndrome
ADAM: A disintegrin and metalloproteinase family of proteinases
ADAMTS: A disintegrin and metalloproteinase with a thrombospondin type 1 motif
ADP: Adenosine diphosphate
AF: Atrial fibrillation
AIS: Acute ischemic stroke
AMI: Acute myocardial infarction
APT: Antiplatelet therapy
ATP: Adenosine triphosphate
Beta-tg: β-thromboglobulin
CAD: Coronary artery disease
CD40L: CD40 ligand
CLEC-2: C-type lectin-like receptor 2
CXCL: CXC ligand
DVT: Deep vein thrombosis
ELISA: Enzyme-linked immunosorbent assay
GP: Glycoprotein
HDL: High-density lipoprotein
HETE: Hydroxyeicosatetraenoic acid
IS: Ischemic stroke
MI: Myocardial infarction
MMP: Metalloproteinase(s)
PAD: Peripheral artery disease
PADGEM: Platelet activation-dependent granule external membrane protein
PAI-1: Plasminogen activator inhibitor 1
PCI: Percutaneous coronary intervention
PDGF: Platelet-derived growth factor
PF4: Platelet factor 4
PG: Prostaglandin
PLA: Platelet-leukocyte aggregates
PMA: Platelet-monocyte aggregates
PNA: Platelet-neutrophil aggregates
PSGL-1: P-selectin glycoprotein ligand-1
RANTES: Regulated upon activation, normal T-cell expressed and presumably secreted
RPR: Residual platelet reactivity
sCD40L: Soluble CD40 ligand
sCLEC-2: Soluble C-type lectin-like receptor 2
SDF-1α: Stromal cell-derived factor-1α
sP-selectin: Soluble P-selectin
TFPI: Tissue factor pathway inhibitor
TGF-β: Transforming growth factor β
TNF: Tumor necrosis factor
TSP-1: Thrombospondin-1
TxA2: Thromboxane A2
TxB2: Thromboxane B2
UFH: Unfractionated heparin
VEGF: Vascular endothelial growth factor
VTE: Venous thromboembolism
VWF: Von Willebrand factor.
